# Prevalence and association of smokeless tobacco use with the development of periodontal pocket among adult males in Dawan Valley, Yemen: a cross-sectional study

**DOI:** 10.1186/s12971-015-0061-8

**Published:** 2015-11-04

**Authors:** Badr Al-Tayar, Mon Mon Tin-Oo, Mohd Zulkarnian Sinor, Mohammed Sultan Alakhali

**Affiliations:** School of Dental Sciences, Universiti Sains Malaysia, Health Campus, 16150 Kubang Kerian, Kelantan Malaysia; Department of Periodontology, Faculty of Dentistry, Jazan University, Jazan, Saudi Arabia

**Keywords:** Periodontal pocket, Community Periodontal Index, Shammah, Smokeless tobacco, Males

## Abstract

**Background:**

The traditional type of smokeless tobacco used in the Arabian Peninsula, particularly common in Yemen, is called shammah. This study aims to determine the prevalence of shammah use and its association with the development of periodontal pockets. Other associated factors with the development of periodontal pocket were also determined.

**Methods:**

This cross-sectional study included 346 adult males aged 18 years old to 68 years old. Socio-demographic characteristics, oral hygiene practices, and shammah use history were surveyed by using a structured interview questionnaire. The clinical assessment for the presence or absence of periodontal pockets was assessed on the basis of community periodontal index. The chi-square test was used to assess significant differences in study groups in terms of the presence of periodontal pockets. Multivariable logistic regression was selected to assess potential associated factors with the development of periodontal pockets.

**Results:**

Among the 346 adult males, 248 (71.7 %), 30 (8.6 %), and 68 (19.7 %) males never used shammah, were former shammah users, and were current shammah users, respectively. The significant associated factors with the development of periodontal pocket were age group (30 years old and above) (Adjusted Odds Ratio (AOR) = 2.03, 95 % CI: 1.13, 3.65; *P* = 0.018), low family income category (AOR = 2.35, 95 % CI: 1.39, 3.99; *P* = 0.001), former shammah user (AOR = 2.66, 95 %: CI: 1.15, 6.15; *P* = 0.022), and current shammah user (AOR = 6.62, 95 %: CI: 3.59, 12.21; *P* = 0.001).

**Conclusions:**

The results revealed that periodontal pockets were significantly associated with age group (30 years old and above), low family income category, former shammah use, and current shammah use. The findings of the current study highlighted the need to develop comprehensive shammah prevention programs and reduce periodontal disease and other shammah-associated diseases.

## Background

Although tobacco cigarette smoking seems common worldwide, smokeless tobacco (SLT) is widely used in various forms, such as in tobacco chewing and snuffing [1]. Studies in South Asia and Southeast Asia have identified that the use of SLT in these regions is similar to betel quid chewing [2] and snuff-dipping or snus habits in the United States and Scandinavia [3]. The traditional type of SLT used in the Arabian Peninsula, including Yemen and several parts of the Kingdom of Saudi Arabia and Algeria, is known as shammah [4–6].

Shammah is a snuff-dipping form of SLT prepared by mixing powdered tobacco, calcium oxide, ash, black pepper, oils, and flavors [7]. This form of SLT is available in several varieties and is grouped according to color and composition. Some forms of shammah are used as white, black, and gray powder [5]. The gray powder is locally known as toombak and is the most commonly used form in South Yemen. Toombak is composed of powdered tobacco leaf and ash.

To date, few studies have been conducted on the prevalence of shammah use in the Arabian Peninsula. Most of these studies focused on the prevalence of shammah use among adolescents rather than among adults [8, 9]. In Yemen, a cross-sectional study was conducted on adult outpatients aged 18 years old and older. These outpatients visited dental clinics at the Al-Thawra Modern General Hospital in Sana’a, Yemen. The study results showed that 4.4 % of the participants (23 patients out of 520 patients) were shammah users [10].

SLT use may cause cancer of the head and neck [11], pancreas [12], and oral diseases such as periodontal disease and mucosal lesions [13, 14]. Periodontal disease is a condition resulting from a complex interaction of many factors, such as age [15], socioeconomic status [16], oral hygiene practice [17], and SLT use [18]. These factors may affect the progression of periodontal disease. However, bacterial plaque is considered the primary etiological factor [19].

The mechanisms in which tobacco use contributes to the pathogenesis of periodontitis are not yet clearly understood. However, the fundamental mechanisms leading to the development of chronic periodontitis are closely related to the dynamics of the immune and inflammatory responses of the host to the periodontal pathogens in the dental biofilm immunoregulator controlling the T helper 1/ T helper 2 cytokine profiles in a periodontal disease [20].

Immune and inflammatory responses are important for understanding the pathogenesis of periodontal diseases, and these responses are orchestrated by a number of either intrinsic or induced host-related factors [21]. A balance between microbial virulence factors and host response exists under normal circumstances. Accordingly, tissue homeostasis is maintained as long as this balance is preserved. The balance between microbial virulence factors and host response in periodontitis is impaired in favor of microbial challenge. Tobacco is an environmental factor that interacts with the host cells and affects the inflammatory responses to this microbial challenge [22]. The toxic components of tobacco (mainly nicotine) may also directly or indirectly cause the deterioration of periodontal tissues.

Nicotine, a major component and the most pharmacologically active agent of tobacco, is likely a significant factor in the exacerbation of periodontal diseases with an increase in pocket depths, loss of periodontal attachment and alveolar bone, and high rate of tooth loss [16]. Previous studies have shown that oral SLT use is associated with gingival and periodontal effects, such as gingival recession [23], gingivitis [9], and interproximal attachment loss [18]. However, the relationship between SLT use and generalized periodontal conditions has not been definitively demonstrated [24]. Furthermore, other studies have revealed that SLT is not associated with periodontal disease [9, 25].

Nonetheless, studies on SLT affecting adult periodontal health, particularly shammah use, have been rarely performed. For the current study, we hypothesize that shammah use is associated with development of periodontal pockets. Therefore, this study aims to improve our understanding of the effect of shammah use on oral health among adult males in Yemen.

## Methods

### Study design and area

A cross-sectional study was conducted from June 2014 to November 2014. A total of 346 participants aged 18 years old to 68 years old were invited to Al-Ebtesammah Dental Clinic in Dawan Valley, Yemen. Dawan Valley is a district of Hadhramout Province located in the eastern part of the Republic of Yemen in the Arabian Sea coast.

### Inclusion and exclusion criteria

Yemeni males aged 18 years old and older with at least two intact natural teeth were included in this study. Smokers, khat leaf chewers, individuals diagnosed with diabetic mellitus, blood dyscrasia, immunodeficiency diseases, mental disorders, and hearing impairment, as well as individuals undergoing periodontal treatment procedures and consuming drugs affecting periodontal status, were excluded from this study.

#### Ethical approval

All of the participants provided written informed consent before they were enrolled in this study. This study was approved by the Ministry of Health and Population in Yemen and the Human Research Ethics Committee at Universiti Sains Malaysia (Ref No: (USM/JEPeM/283.2 (6)/Amend.(01). FWA Reg. No: 00007718; IRB Reg. No: 00004494).

#### Sampling method

**Fig. 1:**
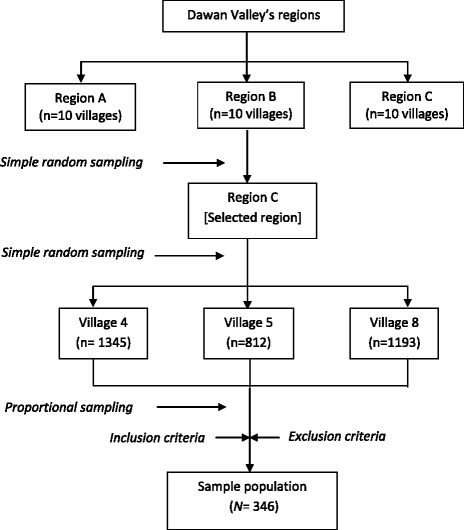
Flow chart of sampling the participants residing in Dawan valley using multi-stage sampling method to obtain the calculated sample size

A multi-stage random sampling technique was used to select the study location. On the basis of the list of the people’s committee in Dawn Valley, a simple random sampling approach was employed to select the participants for this study (Fig. 1).

#### Research questionnaire

A structured questionnaire was designed to record socio-demographic characteristics (age, family income, and education level), oral hygiene practices (daily tooth brushing, daily dental flossing, dental visits, and mouth rinsing with water after shammah use), shammah status (non-user, former user, and current user), frequency of shammah use per day, annual duration of shammah use, and duration of shammah in the mouth per minute. In terms of shammah status, current shammah users were individuals who consumed shammah either daily or occasionally; former users were those who consumed no shammah for at least a year; non-users were those who have never consumed shammah.

#### Oral examination

Before data collection, a calibration exercise was performed to diagnose the periodontal status of 20 shammah users. The kappa score of raters was 0.91 (*P* < 0.001; 95 % CI = 0.74, 1.08).

The WHO Community Periodontal Index (CPI) [26] was employed for the clinical assessment of the presence or absence of periodontal pockets using a CPI probe (no. 621). A participant with periodontal pocket depth 4 or 5 mm were given score 3 and participants with periodontal pocket depth > 6 mm were scored 4. CPI scores 3 – 4 were grouped as presence of periodontal pockets. By contrast, participants who had CPI score 0 (healthy periodontium), score 1 (bleeding observed, directly or by using mouth mirror, after sensing with CPI probe), and score 2 (calculus felt during probing but all the black area of the visible) were group as absence of periodontal pockets.

In CPI, dentition is divided into 6 sextants defined by tooth number. For each sextant, the examined index teeth were 17, 16, 11, 26, 27, 47, 46, 31, 36, and 37, in which the highest score was recorded. A sextant was examined if two or more teeth were present but not subjected to extraction. The participants underwent a clinical oral examination on a dental chair under good lighting conditions. A trained dental assistant was assigned to handle the equipment’s and assist in recording.

#### Statistical analysis

Data entry and analysis were performed by using SPSS 20.0 (IBM, USA). Descriptive data were presented as percentage (%) for categorical variables. The chi-square test was used to assess the significant differences in study groups in terms of the presence of periodontal pockets. Multiple logistic regression analyses was performed to determine the potential factors associated with dependent variable (scores 0–2 of CPI/ scores 3–4 of CPI). The variables were selected and included in the multiple logistic regression analysis model in accordance with the backward step-wise multiple logistic regression method. Multicollinearity and interaction terms were verified. Hosmer–Lemeshow goodness-of-fit test results, classification tables, and area under the receiver operating characteristic curve were obtained to assess the model fitness.

## Results

### Comparison of socio-demographic characteristics of the participants with and without periodontal pockets

Table 1 shows the socio-demographic characteristics of the participants with and without periodontal pockets. A total of 346 participants were analyzed in the current study. The prevalence of periodontal pocket is 26.6 %. Participants 30 years old and above have significantly higher proportions of periodontal pockets (73.9 %) than patients aged 18–29 years old (26.1 %) (*P* = 0.043). The proportion of periodontal pocket is higher (62.0 %) among participants who have a family income of equal to or less than YER 20,000 per month (*P* = 0.001). Participants belonging to the lowest level of education have the highest prevalence of periodontal pockets (54.3 %) (*P* = 0.001).

**Table 1:** Comparison of socio-demographic characteristics, oral hygiene practice, and history of shammah use of periodontal and non-periodontal pockets among adult males in Dawan Valley, Yemen

Variable	Periodontal pocket, *n* (%)		Total	*P* value*
	Absence^a^	Presence^b^		
	254 (73.4)	92 (26.6)		
Socio-demographic characteristics				
Age in years				
18–29	96 (37.8)	24 (26.1)	120 (34.7)	0.043
≥ 30	158 (62.2)	68 (73.9)	226 (65.3)	
Family income (monthly)				
YER More than 20,000	158 (62.2)	35 (38.0)	193 (55.8)	0.001
YER 20,000 or less	96 (37.8)	57 (62.0)	153 (44.2)	
Educational level				
Tertiary	73 (28.7)	10 (10.9)	83 (24.0)	0.001
Non and primary	70 (27.6)	50 (54.3)	120 (34.7)	
Secondary	111 (43.7)	32 (34.8)	143 (41.3)	
Oral hygiene practice				
Tooth brushing/day				
Once or more	111 (43.7)	20 (21.7)	131 (37.9)	0.001
Sometimes	35 (13.8)	17 (18.5)	52 (15.0)	
Never	108 (42.5)	55 (59.8)	163 (47.1)	
Daily dental flossing				
Yes	18 (7.1)	3 (3.3)	21 (6.1)	0.188
No	236 (92.9)	89 (96.7)	325 (93.9)	
Dental attendance				
Regularly or occasionally	41 (16.1)	8 (8.7)	49 (14.2)	0.214
Sometimes	80 (31.5)	32 (34.8)	112 (32.4)	
Never	133 (52.4)	52 (56.5)	185 (53.5)	
Mouth rinse after shammah (*n* = 68)				
Yes	20 (69.0)	32 (82.1)	52 (76.5)	0.208
No	9 (31.0)	7 (17.9)	16 (23.5)	
History of shammah use				
Never shammah user	206 (81.1)	42 (45.7)	248 (71.7)	0.001
Former shammah user	19 (7.5)	11 (12.0)	30 (8.6)	
Current shammah user	29 (11.4)	39 (42.4)	68 (19.7)	
Frequency of shammah per day (*n* = 68)				
1–5 times	10 (34.5)	8 (20.5)	18 (26.5)	0.405
6–10 times	15 (51.7)	23 (59.0)	38 (55.9)	
> 10 times	4 (13.8)	8 (20.5)	12 (17.6)	
Duration of shammah (in year) (*n* = 68)				
1–5 years	18 (62.1)	2 (5.1)	20 (29.4)	0.001
6–10 years	9 (31.0)	12 (30.8)	21 (30.9)	
>10 years	2 (6.9)	25 (64.1)	27 (39.7)	
Duration of shammah placed in mouth (in minute) (*n* = 68)				
1–5 min	8 (27.6)	3 (7.7)	11 (16.2)	0.028
>5 min	21 (72.4)	36 (92.3)	57 (83.8)	

Score 0, Healthy gingivae; score 1, Gingival bleeding after probing; score 2, Calculus detected; score 3, Pocket 4 to 5 mm; score 4, Pocket 6 mm or more

*Chi- Square test was applied

^a^CPI scores 0–2;

^b^CPI scores 3–4

### Comparison of oral hygiene practice of the participants with and without periodontal pocket

Table 1 shows the distribution of the participants by oral hygiene practices, such as daily tooth brushing, flossing, dental visits, and mouth rinse with water after shammah use in relation to the presence of periodontal pockets. In particular, the prevalence of periodontal pockets (59.8 %) is highest among participants who never brush their teeth after shammah use (*P* = 0.001). The participants who never used dental floss for cleaning their teeth have higher proportions of periodontal pockets (96.7 %) than participants who used dental floss (3.3 %). However, the difference is not statistically significant (*P* = 0.188). The prevalence of periodontal pocket is higher (56.5 %) among participants who never had dental visits compared with other categories of dental attendance variables. However, no significant association exists between groups even though the prevalence of periodontal pockets is higher (82.1 %) among participants who rinsed their mouth with water after using shammah compared with those who did not use it.

### Comparison of history of shammah use of the participants with and without periodontal pockets

Of total of 248 participants 71.7 % have never used shammah, 30 participants (8.6 %) are former shammah users, and 68 participants (19.7 %) are current shammah users (Table 1). The proportion of periodontal pockets is 45.7, 12.0, and 42.4 % among non-shammah users, former shammah users, and current shammah users, respectively. Among the shammah users in this research, the participants who used shammah 6–10 times per day have higher proportions of periodontal pocket (59.0 %) than participants in other groups. However, the difference is statistically insignificant. With regard to the annual duration of shammah use, the participants who have been shammah users for more than10 years have higher proportions of periodontal pockets (64.1 %) than participants in other groups. In terms of duration of shammah use placed in the mouth per minute, the participants who placed shammah in their mouths for more than 5 min have higher proportions of periodontal pockets (92.3 %) than participants in other groups (Table 1).

### Risk indicators associated with periodontal pocket

Multiple logistic regression analysis was performed to determine the risk indicators in development of periodontal pockets. Table 2 shows significant association between periodontal pocket development and age. Participants aged 30 years old and above have 2.03 times higher odds of developing periodontal pocket than participants aged 18–29 years when other variables are adjusted. Moreover, participants with family income of YER 20,000 or fewer have 2.35 times higher odds of developing periodontal pockets than participants with higher family income (more than YER 20,000). Participants who previously used shammah have 2.66 times higher odds of developing periodontal pocket than those participants who have never used shammah. Participants who currently use shammah are 6.62 times more likely to develop periodontal pocket than participants who have never used shammah.

**Table 2:** Factors associated with periodontal pocket among adult males in Dawan valley, Yemen by multiple logistic regression analyses

Variable	COR^a^	*P* **-** value^a^	AOR^b^	*P* **-** value^b^
	(95 % CI)		(95 % CI)	
Socio-demographic characteristics				
Age in years				
18–29	1.00		1.00	
≥ 30	1.72 (1.01, 2.93)	0.045	2.03 (1.13, 3.65)	0.018
Family income				
YER More than 20,000	1.00		1.00	
YER 20,000 or less	2.68 (1.64, 4.38)	0.001	2.35 (1.39, 3.99)	0.001
Shammah status				
Never shammah user	1.00		1.00	
Former shammah user	2.84 (1.25, 6.40)	0.001	2.66 (1.15, 6.15)	0.022
Current shammah user	6.59 (3.67,11.82)	0.001	6.62 (3.59,12.21)	0.001

- ^a^Simple logistic regression, ^b^Multiple logistic regression

- Backward step wise LR multiple logistic regression was applied

- Multicolinearity and interaction term were checked and did not found

- Hosmer-Lemeshow test, (*P* = 0.865), classification table (overall correctly classified percentage (78.3 %) and area under Receiver Operating Characteristics (ROC) curve (74.1 %) were checked the fit of the model and reported to be fit

## Discussion

Population studies on the prevalence of SLT use in the Arabian Peninsula are scarce [8, 10]. In this study, the prevalence of SLT use (19.7 %) among adult males is comparable to previous findings in India (21 %) [10]. However, this value is higher than the reported value in Saudi Arabia (2.0 %) [8]. High prevalence of SLT use in this study can be attributed to the rural nature of Dawan Valley. Previous studies have reported high prevalence rates of SLT use in mountainous and rural areas [27–29].

Periodontal disease has emerged as a major public health problem and is among the most prevalent chronic diseases leading to tooth loss [30]. Such diseases are serious threats to the prevention of oral diseases in developed and developing countries, including Yemen. Data describing the risk factors associated with periodontal disease, particularly shammah are highly limited in Yemen.

The prevalence of periodontitis in this study is comparable with a study conducted in China (25.9 %) [31]. However, a higher prevalence of periodontitis (44.69 %) has been recorded in Libya [32], but a lower prevalence (15.8 %) was reported among adults in India [33] than that obtained in the present study. This difference can be attributed to sampling size and methods in the studies.

The results of multiple logistic regression analysis reveal that shammah use is a factor that influences the development of periodontal pockets after socio-demographic characteristics and oral hygiene practices have been adjusted. Current shammah users are at greater risk of developing periodontal pocket than former and non-shammah users. The onset of periodontal pockets differs considerably between current and former shammah users. Thus, we conclude that the previous and current use of shammah plays an analogous role in the disappearance of periodontal disease once the shammah use is stopped. Our findings are similar to those of previous studies that report a significant association between periodontal disease and tobacco use [18, 34, 35]. Kumar et al. [36] examined 513 dentate adult males to determine the effect of tobacco use on the severity of periodontal disease in India. Their study showed that SLT users are likely to develop periodontal disease than smokers. By contrast, a study conducted in Saudi Arabia revealed that shammah is not a significant risk indicator in development of periodontal disease [9]. Such contradictory observations may be attributed to several factors, such as differences in the trends of oral SLT practices and in the type of SLT products used by the respective populations.

Previous studies have supported the strong confounding effects of socio-demographic factors on periodontal pocket development [37, 38]. Eke et al. [39] estimated the prevalence, severity, and extent of periodontitis on a sample of 3742 adults aged 30 years old and above. Their study showed that periodontitis increases with age. The study of Gundala and Chava [40] shows a significant decrease in periodontitis with increasing income. Similarly, the current study highlights the association of age and lower family income with periodontal disease development.

## Conclusion

The prevalence of SLT use is (19.7 %). Using shammah for more than 10 years and placing the shammah in the mouth for more than 5 min are associated with periodontal pockets. Current shammah users as well as former shammah users, individuals age older than 30 years, and low family income group are at high risk in development of periodontal pockets. The findings of the current study highlight the need to develop comprehensive shammah prevention programs and reduce periodontal disease and other shammah-associated diseases.

## Abbreviations

AOR: adjusted odds ratio
CI: confidence interval
COR: crude odds ratio
CPI: community periodontal index
SD: standard deviation
SLT: smokeless tobacco
U.S.: United States
YER: Yemeni Riyal

## References

[1] Layten Davis D, Nielsen MT (1999). Tobacco: production, chemistry and technology.

[2] Gupta P, Ray C (2004). Epidemiology of betel quid usage. Ann Acad Med Singapore..

[3] Axéll TE (1993). Oral mucosal changes related to smokeless tobacco usage: research findings in Scandinavia. Eur J Cancer B Oral Oncol.

[4] Zhang X, Schmitz W, Gelderblom H, Reichart P (2001). Shammah—induced oral leukoplakia-like lesions. Oral Oncol.

[5] Scheifele C, Nassar A, Reichart P (2007). Prevalence of oral cancer and potentially malignant lesions among shammah users in Yemen. Oral Oncol.

[6] Alsanosy RM (2013). Smokeless tobacco (shammah) in saudi arabia: a review of its pattern of use, prevalence, and potential role in oral cancer. Asian Pac J Cancer Prev.

[7] Samman MA, Bowen ID, Taiba K, Antonius J, Hannan MA (1998). Mint prevents shamma-induced carcinogenesis in hamster cheek pouch. Carcinogenesis.

[8] Al Agili DE, Park HK (2012). The prevalence and determinants of tobacco use among adolescents in Saudi Arabia. J Sch Health.

[9] Al Agili D, Park H (2013). Oral health status of male adolescent smokeless tobacco users in Saudi Arabia. East Mediterr Health J.

[10] Hajeb R. Prevalence of oral mucosal lesions and related risk habits in outpatient dental clinics in Malaysia and Yemen. 2010. http://studentsrepo.um.edu.my/3670/2/SECOND_PART._DR._RAJI_MANSOOR._DISSERTATION.pdf. Accessed 22 Feb 2015.

[11] Zhou J, Michaud DS, Langevin SM, McClean MD, Eliot M, Kelsey KT (2013). Smokeless tobacco and risk of head and neck cancer: Evidence from a case–control study in New England. Int J Cancer.

[12] Boffetta P, Aagnes B, Weiderpass E, Andersen A (2005). Smokeless tobacco use and risk of cancer of the pancreas and other organs. Int J Cancer.

[13] Singh G, Rizvi I, Gupta V, Bains VK. Influence of smokeless tobacco on periodontal health status in local population of north India: A cross-sectional study. Dent Res J. 2011; doi:10.4103/1735-3327.86045. 10.4103/1735-3327.86045 PMC3221089 22135693

[14] Lesan S, Nosratzehi T, Ousia M, Arbabikalati F, Pourmardan E (2014). The Correlation between the Frequency of Oral Lesions and the Amount of Smokeless Tobacco Usage in Patients Referred to Oral Medicine Department of Zahedan Dental School. J Dent (Shiraz).

[15] Mahmud SZ, Alif SM, Tarafder MA, Hossain SM. The Correlation between Periodontal Diseases and Chronological Age among Type 2 Diabetes Mellitus Patients attending at National Healthcare Network (NHN) Mirpur Centre, Dhaka, Bangladesh. Birdem Med J. 2013. http://banglajol.info/index.php/BIRDEM/article/view/17209

[16] Kamath DG, Verma B, Kamath S (2012). Effect Of Socioeconomic Status On Periodontal Health Of a Population in Mangalore. Natl J Integr Res Med.

[17] Hopcraft MS, Morgan MV, Satur JG, Wright F, Darby IB. Oral hygiene and periodontal disease in Victorian nursing homes. Gerodontology. 2012; doi:10.1111/j.1741-2358.2010.00448.x. 10.1111/j.1741-2358.2010.00448.x 21083744

[18] Fisher M, Taylor G, Tilashalski K (2005). Smokeless tobacco and severe active periodontal disease, NHANES III. J Dent Res..

[19] Listgarten M. The role of dental plaque in gingivitis and periodontitis. J Clin Periodontol. 1988; doi:10.1111/j.1600-051X.1988.tb01019.x. 10.1111/j.1600-051x.1988.tb01019.x 3053789

[20] Gemmell E, Seymour GJ (2004). Immunoregulatory control of Th1/Th2 cytokine profiles in periodontal disease. Periodontology 2000.

[21] Taubman MA, Valverde P, Han X, Kawai T (2005). Immune response: the key to bone resorption in periodontal disease. J Periodontol..

[22] Palmer RM, Wilson RF, Hasan AS, Scott DA (2005). Mechanisms of action of environmental factors–tobacco smoking. J Clin Periodontol..

[23] Montén U, Wennström JL, Ramberg P (2006). Periodontal conditions in male adolescents using smokeless tobacco (moist snuff). J Clin Periodontol.

[24] Wouters F, Salonen L, Frithiof L, Hellden L (1993). Significance of some variables on interproximal alveolar bone height based on cross‐sectional epidemiologic data. J Clin Periodontol.

[25] Kallischnigg G, Weitkunat R, Lee PN (2008). Systematic review of the relation between smokeless tobacco and non-neoplastic oral diseases in Europe and the United States. BMC Oral Health.

[26] World Health Organization. Oral health survey-basic method 4th edition. 1997. http://www2.paho.org/hq/dmdocuments/2009/OH_st_Esurv.pdf. Accessed 22 May 2015.

[27] Neufeld K, Peters D, Rani M, Bonu S, Brooner R (2005). Regular use of alcohol and tobacco in India and its association with age, gender, and poverty. Drug Alcohol Depend.

[28] Sreeramareddy CT, Ramakrishnareddy N, Kumar H, Sathian B, Arokiasamy J. Prevalence, distribution and correlates of tobacco smoking and chewing in Nepal: a secondary data analysis of Nepal Demographic and Health Survey-2006. Subst Abuse Treat Prev Policy. 2011; doi:10.1186/1747-597X-6-33 10.1186/1747-597X-6-33 PMC3266635 22185233

[29] Agbor M, Azodo C, Tefouet T. Smokeless tobacco use, tooth loss and oral health issues among adults in Cameroon. Afr Health Sci. 2013; doi:10.4314/ahs.v13i3.38. 10.4314/ahs.v13i3.38 PMC3824433 24250322

[30] Petersen PE, Ogawa H. The global burden of periodontal disease: towards integration with chronic disease prevention and control. Periodontol 2000. 2012; doi:10.1111/j.1600-0757.2011.00425.x 10.1111/j.1600-0757.2011.00425.x 22909104

[31] Zhang Q, Li Z, Wang C, Shen T, Yang Y, Chotivichien S et al. Prevalence and predictors for periodontitis among adults in China, 2010. Glob Health Action. 2014. doi:10.3402/gha.v7.24503. 10.3402/gha.v7.24503 PMC4090366 25008055

[32] Peeran SW, Singh AR, Alagamuthu G, Kumar PN (2013). Periodontal status and its risk factors among young adults of the Sebha city (Libya). Dent Res J (Isfahan).

[33] Kumar S, Dagli RJ, Dhanni C, Duraiswamy P (2009). Relationship of body mass index with periodontal health status of green marble mine laborers in Kesariyaji. India. Braz Oral Res..

[34] James JA, Sayers NM, Drucker DB, Hull PS (1999). Effects of tobacco products on the attachment and growth of periodontal ligament fibroblasts. J Periodontol.

[35] Mohamed S, Janakiram C (2013). Periodontal status among tobacco users in Karnataka. India. Indian J Public Health..

[36] Kumar S, Dagli RJ, Chandrakant D, Prabu D, Suhas K (2008). Periodontal status of green marble mine labourers in Kesariyaji, Rajasthan. India. Oral Health Prev Dent..

[37] Yalcin F, Eskinazi E, Soydinc M, Basegmez C, Issever H, Isik G (2002). The effect of sociocultural status on periodontal conditions in pregnancy. J Periodontol.

[38] Dhaliwal JS, Lehl G, Sodhi SK, Sachdeva S. Evaluation of socio-demographic variables affecting the periodontal health of pregnant women in Chandigarh, India. J Indian Soc Periodontol. 2013; doi:10.4103/0972-124X.107475 10.4103/0972-124X.107475 PMC3636946 23633773

[39] Eke P, Dye B, Wei L, Thornton-Evans G, Genco R (2012). Prevalence of periodontitis in adults in the United States: 2009 and 2010. J Dent Res.

[40] Gundala R, Chava VK. Effect of lifestyle, education and socioeconomic status on periodontal health. Contemp Clin Dent. 2010; doi:10.4103/0976-237X.62516 10.4103/0976-237X.62516 PMC3220063 22114373

